# Prevalence of Typhoid Fever in Chicago

**Published:** 1882-05

**Authors:** 


### ARTICLE VII.

### CHICAGO MEDICAL SOCIETY.

Stated meeting, April 17, 1882. Dr. J. H. Hollister, President in the chair.

After the reading of the minutes of the last meeting, by Dr. L. H. Montgomery, secretary, Dr. Hollister read a carefully prepared paper, on the recent prevalence of typhoid fever in Chicago. The questions briefly discussed were, (*a*) What is Typhoid Fever? (*b*) How is it developed? (*c*) What is the manner of its propogation? (*d*) How may we account for its recent prevalence in Chicago? (*e*) What means are available for its control?

Typhoid fever was not, as its name implied, closely allied to Typhus fever, although it was more than a constitutional disturbance caused by a local lesion, and the whole system seemed to suffer from the presence of a specific typhoid poison, which also accompanied anatomical lesions through its entire course.

The lesions in the disease might be divided into two groups: The first seemed to represent the more or less direct effect of the typhoid poison as found in Peyer's patches, in the solitary glands, in the mesenteric glands and in the spleen, involving the lymphatics of the intestinal canal. In the second group, those affections were found which resulted from the general disease. These were mainly the parenchymatous degeneration which in extent and intensity, depended upon the severity and the continuance of the general disease, notably the fever. These degenerations were found most frequently in the liver, the kidneys, the voluntary muscles, and the muscles of the heart, in the brain and the lungs, and the mucous membrane may also be the seat of tissue degeneration if the case were long continued and severe. The lesions of the intestine had been a matter of constant and interesting study. The agminated glands of Peyer became involved as though the specific poison at this particular portion of the alimentary canal sought entrance to the general circulation and here first produced its characteristic effects.

The author of the paper, here exhibited some well-prepared specimens, and described with minuteness the pathological changes that occurred during a four weeks' course of the disease, *i. e.*, where cicatrization was perfected and convalescence had been established. The decomposition of organic matter undoubtedly contributed more to originate the disease than any other single cause, but it must be a specific form of poisoning from animal matter, and especially dependent upon the decomposition of feculent discharges of human product. Filth and decomposition tended strongly to intensify the disease once developed, and the same was true of defective sewerage.

Regarding the manner in which typhoid fever was propagated, it was not disseminated by simple contact with persons having the disease, and therefore it must develop from a miasm outside the human body. In superficial vaults and cesspools the specific poison might multiply itself to an incredible extent, infecting the earth, the air and water, and the vitality of the poison could maintain itself for a longer time than could the specific poison producing cholera.

During the last eight months, 572 deaths from typhoid fever occurred in Chicago—in Cook County Hospital, where 100 cases were treated, 16 died. This ratio made the probable number of cases of typhoid fever in the city during the last eight months, 3,575. The fifth, sixth, ninth and fourteenth wards had by far the largest representation. The fatality of typhoid fever gave warning of what was in store for us if the sanitary conditions of the city were allowed to remain as they were—and the means available for the control of the disease, were for the authorities to see that the tunnels were kept free from contamination with drain and shore pollutions; and the sewers must be looked to. The Illinois and Michigan Canal should be utilized for the drainage of Chicago.

Dr. N. S. Davis remarked that there had been 143 deaths from typhoid in eight months, from April to December, 1880; that there had been 520 deaths from the same cause in the corresponding months in 1881. The Doctor highly recommended iodine in the treatment of typhoid fever.—See the JOURNAL for February, 1882.

Possibly we had in iodine a general alterative, not depressing in character, and also an antiseptic. Bromine, he had used years ago; it actually diminished the disease, but it was difficult of administration on account of its pungency. He considered iodine the leading remedy. He used it for the last six months in hospital and private practice, together with some other remedies, turpentine emulsion, sponging, the wet sheet, etc. In 27 cases, one died, but the disease had become complicated by broncho-pneumonia and laryngitis. The Doctor gave iodine in the following manner:

℞ Iodini.......................................gr. viii.

Potassii iodidi.................................gr. xxx.

M. Aquæ distil............... ..................... ℥jss.

S. 12 to 15 drops in sweetened water every three hours, for three or four consecutive days.

Dr. J. G. Kiernan read a paper on the occipital lobe in relation to intelligence, in which he called attention to the fact that the importance of the frontal lobe has been much over-estimated. He claimed that in the occipital lobe were to be found the great ssociation centers of the brain, and as upon these depended man's intelligence, the full development of the occipital lobe was necessary to constitute a well-balanced cerebral system. He cited as corroboratory evidence the fact that the occipital lobes occur only in the primates, being absent even in the lower monkeys, whereas frontal lobes are found in all mammalia, and the fact that the occipital lobes are the last to make their appearance in the fœtus. The occipital lobe was markedly deficient in the reasoning maniacs. In this connection the cast of Guiteau's head was exhibited, which was described as being very exact, and upon inquiry, Dr. Kiernan stated that Guiteau's skull is three times the usual amount of asymmetry.

Dr. D. R. Brower followed with some interesting remarks, in which he quoted Dr. Gray, of the Utica Asylum, as having stated as an expert, at the trial of Guiteau in Washington, that the criminal's skull was of a normal asymmetry.

